# Integrated transcriptomic and metabolomic analyses of early defense responses in resistant and susceptible sweet potato cultivars under *Cylas formicarius* herbivory

**DOI:** 10.3389/fpls.2026.1901251

**Published:** 2026-08-18

**Authors:** Youmiao Li, Henan Ju, Wanqiu Huang, Huifeng Li, Yongmei Huang, Yanqing Li, Zhenwei Li, Xialin Zheng, Jinfeng Hua

**Affiliations:** 1 Department of Sweet Potato Genetic Breeding and Application, Institute of Maize Research, Guangxi Academy of Agricultural Sciences, Nanning, China; 2 College of Agriculture, Guangxi University, Nanning, China

**Keywords:** sweet potato, *Cylas formicarius*, transcriptomics, metabolomics, insect resistance mechanism, WGCNA

## Abstract

**Introduction:**

Sweet potato weevils (*Cylas formicarius*) are devastating pest that seriously affect sweet potato production, and their feeding can significantly reduce sweet potato yield and quality.

**Methods:**

To elucidate the molecular mechanisms underlying sweet potato responses to *C. formicarius* herbivory, the resistant cultivar J2 and the susceptible cultivar G12 were selected for integrated transcriptomic and metabolomic analyses of leaves and storage roots during the early stage of infestation.

**Results:**

The results demonstrated that, in storage roots, the resistant J2 exhibited a greater number of differentially expressed genes (DEGs) and differentially accumulated metabolites (DAMs) than G12; however, in leaves, J2 displayed more DEGs but fewer DAMs than G12, and these were primarily enriched in defense-related pathways, including phenylpropanoid biosynthesis, amino acid biosynthesis, flavonoid biosynthesis, and α-linolenic acid metabolism. Integrated multi-omics analysis further suggested that phenylpropanoid metabolism and amino acid biosynthesis may represent key regulatory pathways involved in sweet potato responses to *C. formicarius* herbivory. Weighted gene co-expression network analysis (WGCNA) identified candidate modules and hub genes significantly associated with resistance to *C. formicarius*, including key phenylpropanoid biosynthesis genes such as *PAL*, *C4H*, and *PPO*.

**Discussion:**

These results suggest that the resistant cultivar J2 enhances is associated with defense against *C. formicarius* by activating phenylpropanoid and flavonoid metabolism, upregulating JA-associated signaling, and coordinating physical barrier formation with chemical defense accumulation. This study provides a theoretical basis and potential targets for molecular breeding and candidate gene mining in insect-resistant sweet potato.

## 1 Introduction

Sweet potato [*Ipomoea batatas* (L.) Lam.] plays a crucial role in ensuring global food security and increasing farmers’ income as an important food crop, feed source, industrial raw material, and energy crop (Mwanga et al., 2021; Salelign and Duraisamy, 2021; Mounika et al., 2024). According to statistics from the Food and Agriculture Organization of the United Nations (FAO), the global sweet potato planting area reached 7.570 × 10^6^ hm² in 2023, with a total production of approximately 9.352 × 10^7^ t. The main production areas are concentrated in China, Malawi, Nigeria, Tanzania, Uganda, and Indonesia (FAO, 2026). With the rapid development of the sweet potato industry, market demand continues to grow. However, driven by global warming, agricultural migration, and the expansion of international trade, the increasing occurrence and expanding damage range of *Cylas formicarius* Fabricius (Coleoptera: Brentidae) have become one of the key biotic stresses constraining the sustainable development of the sweet potato industry (George et al., 2024; Junaid and Gokce, 2024).

Larvae of *C. formicarius* tunnel into storage roots, causing rot, foul odor, and loss of edible value, while adults feed on stems, leaves, and storage roots, impairing plant growth and facilitating disease (Rehman et al., 2019; Keyser et al., 2024). Sweet potato is grown in 109 countries, over 80 of which are affected by this pest, with yield losses ranging from 30% to 100% (Liu et al., 2022; Ngcobo et al., 2024). The damage continues to expand, threatening the sustainability of the sweet potato industry (Baro et al., 2022; Xu et al., 2025). Although chemical, agricultural, pheromone-based, and biological controls are commonly used, they face problems such as pesticide residues, resistance, the cryptic habit of the pest, and inconsistent field efficacy (Hua et al., 2026; Li et al., 2026). Developing resistant cultivars offers a cost-effective, ecologically safe, and sustainable alternative. Therefore, elucidating the genetic and metabolic basis of resistance to *C. formicarius* is essential for breeding resistant cultivars and establishing integrated management systems (Schloemer et al., 2025).

Unlike mobile organisms, plants are sessile and inherently incapable of evading environmental stresses through physical relocation. Consequently, over prolonged evolutionary periods, they have developed complex defense mechanisms to counteract herbivory by various phytophagous insects (Meents et al., 2019; Ahmed et al., 2024). Plant defense against herbivorous insects typically includes constitutive defense and inducible defense (Bohloolzadeh et al., 2024; Pastierovic et al., 2024a). Constitutive defense mainly depends on epidermal waxes, the degree of suberization, tissue hardness, or the production of toxic chemicals; inducible defenses are rapidly activated following insect herbivory, mediated primarily by signaling networks involving jasmonic acid (JA), ethylene (ET), and salicylic acid (SA), further regulating the synthesis and accumulation of defense-related secondary metabolites, such as phenylpropanoids, flavonoids, terpenoids, alkaloids, and protease inhibitors (Khokhar et al., 2023; Zheng et al., 2024). Recent studies have shown that phenylpropanoid and flavonoid metabolism pathways are closely associated with cell wall reinforcement, antioxidant defense, and signal transduction. These pathways also contribute to plant resistance by deterring feeding, inhibiting digestion, and interfering with insect growth and development. In addition, α-linolenic acid metabolism and the downstream JA biosynthetic pathway are widely regarded as key signaling hubs in plant responses to chewing insect herbivores (Erb and Reymond, 2019; Ramaroson et al., 2022; Wang et al., 2024).

With advances in high-throughput sequencing and metabolomics, integrated multi-omics analysis has become an effective approach for elucidating the mechanisms underlying plant-insect interactions (Pandita et al., 2021). Integrating transcriptomic DEGs with metabolomic DAMs enables the identification of major metabolic shifts and potential regulatory pathways in plants under stress. This approach also facilitates the construction of gene-metabolite association networks, thereby providing insight into the molecular basis of plant stress responses (Wang et al., 2021). In maize, integrated transcriptomic and metabolomic analyses revealed significant accumulation of phenylpropanoids and benzoxazinoid metabolites in insect-resistant lines after *Spodoptera exigua* feeding, while the expression of related biosynthetic genes was simultaneously upregulated (Luo et al., 2025). In rice, combined transcriptomic and metabolomic analyses revealed defense responses induced by rice borer oviposition, showing that oviposition stress altered egg survival through the activation of multiple defense-related metabolic pathways (Chen et al., 2025). These recent findings corroborate the unique advantages of multi-omics combined analysis in revealing the deep mechanisms of plant-insect interactions. However, studies investigating sweet potato responses to *C. formicarius* feeding have thus far relied primarily on single-omics approaches. Nokihara et al. identified differentially expressed resistance-related genes through transcriptomic analysis of susceptible cultivars, but these findings were not validated at the metabolite level (Nokihara et al., 2021). Lei et al. performed sRNA sequencing and transcriptome association analysis using storage root tissues, revealing miRNA expression profiles; however, metabolomic data were not incorporated (Lei et al., 2022). Current research on sweet potato responses to *C. formicarius* feeding remains largely limited to single-omics analyses and lacks a systematic investigation of the relationship between transcriptional changes and metabolic reprogramming. Moreover, the coordinated organ and developmental stage-specific defense mechanisms of leaves and storage roots during the resistance response remain poorly understood. Therefore, employing an integrated multi-omics approach is essential to systematically elucidate the response mechanisms of sweet potato to *C. formicarius* herbivory at both transcriptional and metabolic levels. In contrast, the present study integrates transcriptomic and metabolomic analyses in both leaves and storage roots during the early stage of weevil feeding. This integrated approach allows us to systematically link transcriptional reprogramming with metabolic shifts and to identify organ and developmental stage-specific defense strategies, something not achieved in previous sweet potato weevil studies. Moreover, we employ weighted gene co-expression network analysis (WGCNA) to pinpoint hub genes and modules associated with resistance.

In this study, the sweet potato cultivars J2 and G12 were used to compare transcriptomic and metabolomic changes in leaves and storage roots under feeding stress. Through integrated pathway enrichment analysis and WGCNA, key pathways, metabolites, and candidate genes associated with resistance to *C. formicarius* were identified. These findings provide a foundation for elucidating the molecular basis of sweet potato resistance to *C. formicarius*, understanding the coordinated defense responses of aboveground and belowground tissues, and facilitating the molecular breeding of insect-resistant sweet potato cultivars.

## 2 Materials and methods

### 2.1 Insects

*C. formicarius* was collected from sweet potato fields at Mingyang, Guangxi Academy of Agricultural Sciences, Nanning, Guangxi Zhuang Autonomous Region, China, and was reared on fresh sweet potato storage roots at 27 ± 1 °C, and 70 ± 5% relative humidity (RH), and a photoperiod of 16:8 (L: D) h.

### 2.2 Investigation of sweet potato cultivars and their susceptibility to damage by *C. formicarius*

A field experiment was conducted in Mingyang, Nanning, Guangxi, China (22.60°N, 108.23°E), using 100 collected sweet potato cultivars. The experiment followed a randomized complete block design with three replications per cultivar. For each replication, 200 leaves, 40 stems, and 50 storage roots were randomly sampled, and C. *formicarius* damage grades were assessed separately for each plant part. The severity of damage to stems and leaves was classified into six grades: grade 0, no damage; grade 1, slight damage to stems and leaves; grade 2, minor damage to stems and leaves; grade 3, partial damage to stems and leaves; grade 4, severe damage to stems and leaves without plant wilting; grade 5, extensive damage to stems and leaves. Storage root damage grade determination criteria: grade 0, no damage; grade 1, slight damage to the storage root; grade 2, minor damage to the storage root; grade 3, partial damage to the storage root; grade 4, severe damage to the storage roots; grade 5, extensive damage to the storage root. The damage index (DI,%) was calculated as $\text{DI}=(\sum_{\text{i}=0}^{5}{\text{n}}_{\text{i}}\times {\text{v}}_{\text{i}})/(\text{N}\times {\text{v}}_{\text{max}})\times 100$, where n_i_ is the number of damages at grade i, v_i_ is the grade value (0–5), N is the total number of evaluated leaves, stems, and storage roots, and v_max_= 5. A lower damage index indicates higher pest resistance of sweet potato cultivars. To make the damage index visually convertible to pest resistance, the damage index was converted to the damage index reduction rate. Damage index reduction rate (DIR,%) was also calculated as $\text{DIR}=({\text{DI}}_{\text{C}}-{\text{DI}}_{\text{T}}){\text{/DI}}_{\text{C}}\times 100$, where DIC is the average damage index of the control cultivar, and DIT is the average damage index of the test cultivar.

### 2.3 Laboratory assessment of susceptible cultivars

To assess the resistance of sweet potato leaves to *C. formicarius*, 15 male and 15 female adult *C. formicarius* at the same developmental stage (total 30) were placed in net bags and subjected to starvation treatment for 24 hours. The starved adults were then fed on the third leaf of a 30-day-old sweet potato plant for 2, 4, 6, and 8 hours, respectively. Leaves without insect inoculation served as controls. After feeding, the feeding area was calculated using ImageJ software (http://imagej.nih.gov/ij/).

To evaluate the resistance of sweet potato storage roots to *C. formicarius*, adult insects at the same developmental stage were collected and separated into net bags, with 250 females and 250 males per bag (500 insects in total), and starved for 24 h prior to the feeding assay. The pre-starved adults were then transferred to Petri dishes containing 5 g of sweet potato storage root and fed for 2, 4, 6, and 8 h. Sweet potato storage roots without insect infestation were used as the control. After the feeding period, the weights of the storage roots in both the control and insect-fed treatments were recorded, and insect feeding amount was calculated accordingly. Because preliminary tests showed that individual adults consumed very small amounts of root tissue, we used 500 adults per 5 g root sample to ensure that feeding damage could be accurately quantified within the short experimental period (2–8 h) http://imagej.nih.gov/ij/.

For the determination of oviposition preference and larval development inhibition in storage roots, the same batch of storage roots with uniform size and intact skin were selected. Each storage root was placed in a net bag containing 50 adult *C. formicarius* (25 males and 25 females). Adult *C. formicarius* were removed after 7 days of treatment at 28 °C under an 8 h light/16 h dark cycle, and the net bags were maintained under the same conditions for another 30 days. The number of newly emerged adults was recorded daily until no new adults appeared, and emerged adults were removed promptly to prevent re-oviposition.

### 2.4 Sweet potato feeding treatment

The susceptible cultivar Guishu 12 (G12) and the resistant cultivar Jiyuan 2 (J2) were selected for the feeding bioassay. Seedlings with uniform growth were selected and subjected to either indoor or outdoor pot culture. For indoor culture, seedlings were planted in round plastic pots (12 cm diameter) filled with substrate soil and placed in a growth chamber at 28 °C with a 10 h light/14 h dark photoperiod for 30 days. For outdoor culture, seedlings were planted in round grow bags (25 cm × 25 cm), and storage roots were harvested after 120 days.

**Figure 1:**
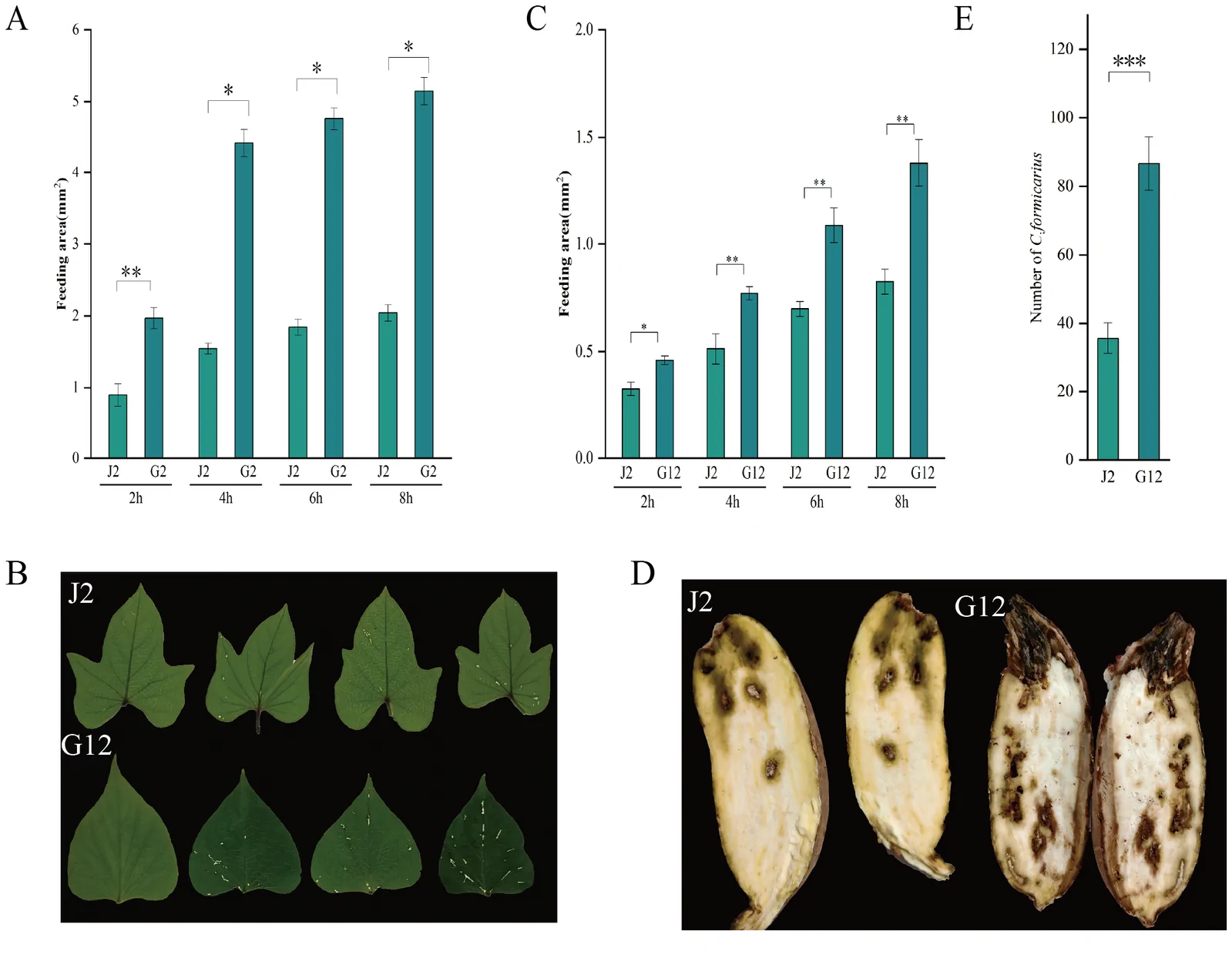
Resistance evaluation of two sweet potato cultivars to *C. formicarius*. **(A)** Leaf feeding area at 2, 4, 6, and 8 h after exposure to adults (n = 3 leaves per cultivar per time point). **(B)** Representative images of leaves after 8 h of feeding. **(C)** Storage root feeding weight at 2, 4, 6, and 8 h after exposure to adults. Values in bars represent the mean feeding amount per adult. Three storage roots per cultivar per time point were used as biological replicates (n = 3). **(D)** Representative images of storage roots after 30 days of infestation. **(E)** Total number of newly emerged adults from storage roots (n = 6 storage roots per cultivar). Fifty adults (1:1 male-to-female ratio) were introduced and removed after 7 days, followed by a 30-day breeding period. Statistical analysis: Data in **(A, C)** were analyzed using two-way analysis of variance (ANOVA) followed by Tukey’s *post-hoc* test; data in **(E)** were analyzed using independent Student’s t-test. All data are presented as mean ± SD. Significance indicators: *P < 0.05, **P < 0.01, ***P < 0.001.

To determine the appropriate sampling time for capturing early defense responses, we first analyzed the kinetics of leaf feeding area and the consumption weight of storage roots. As shown in **Figure 1A**, the feeding area increased sharply within the first 2 h and continued to rise significantly by 4 h, after which the increase slowed markedly between 6 h and 8 h. The root consumption weight increased sharply within the initial 2 hours and continued to rise significantly up to the 4-hour time point, after which the rate of increase markedly slowed during the subsequent 6–8 hour period (**Figure 1C**). These results indicate that the initial 4 h represent a critical window for active herbivory and early defense induction. Accordingly, 0 h and 4 h were selected as the sampling time points for subsequent transcriptomic and metabolomic analyses. To ensure comparability across multi-omics datasets, identical biological samples were used for both transcriptomic and metabolomic profiling. For each combination of cultivar, time point, and tissue type, three biological replicates were collected. Each replicate was divided into two portions: one was immediately frozen in liquid nitrogen for RNA extraction and transcriptome sequencing, and the other was vacuum freeze-dried for metabolite extraction prior to UPLC-MS/MS analysis.

Leaf treatment: after 30 days of indoor cultivation, 30 adult *C. formicarius* (15 males and 15 females) that had been starved for 24 h were introduced onto each seedling. The experiment consisted of non-feeding control (0 h) and *C. formicarius* feeding treatment (4 h), Leaf samples were collected immediately before insect introduction (J0 and G0) and after 4 h of feeding (J4 and G4). with three biological replicates for each treatment of each cultivar. Three fresh young leaves from each plant were pooled as one sample and immediately stored in liquid nitrogen for transcriptome sequencing and metabolome sequencing.

Storage root treatment: 12 fresh sweet potato storage roots of similar size were selected from the storage roots harvested from outdoor cultivation, and each storage root was placed separately in a 20 cm × 20 cm net bag. Each bag contained 50 adult *C. formicarius* (25 males and 25 females) that had been starved for 24 hours. The experiment consisted of non-feeding control (0 h) and *C. formicarius* feeding treatment (4 h). Root samples were collected before insect exposure (J0 and G0) and after 4 h of feeding (J4 and G4). After each sample, each storage root was immediately cut into small pieces and mixed evenly as a single sample and stored in liquid nitrogen for subsequent RNA extraction, metabolite extraction, and other analyses. Three replicates were set for each cultivar at each time point.

In subsequent transcriptomic and metabolomic analyses, feeding-responsive differentially expressed genes (DEGs) and differentially accumulated metabolites (DAMs) were identified through pairwise comparisons of J4 vs. J0 and G4 vs. G0, performed separately for leaf and storage root samples.

This study focused on the early induced response of sweet potato to *C. formicarius* feeding stress; therefore, 0 h and 4 h were selected as the omics sampling time points. Because plant resistance responses exhibit distinct temporal dynamics, the results of this study mainly reflect transcriptional and metabolic changes during the early stage of feeding. Further analyses with additional time points will be needed to characterize the dynamic defense process.

### 2.5 Transcriptome sequencing and analysis

Total RNA was extracted from sweet potato samples using the Plant Total RNA Extraction Kit (Tiangen, Beijing, China), and then the RNA concentration and purity were measured using the NanoDrop 2000 (Thermo Fisher Scientific, Wilmington, DE). RNA integrity was evaluated using the RNA Nano 6000 detection kit from the Agilent Bioanalyzer 2100 system (Agilent Technologies, CA, USA). Sequencing libraries were constructed using the Hieff NGS Ultima Dual-mode mRNA Library Prep Kit for Illumina Yeasen Biotechnology (Shanghai) Co., Ltd. The constructed libraries were sequenced using the Illumina NovaSeq platform (PE150 mode sequencing) with a sequencing read length of 150 bp. The transcriptome library was prepared and sequenced by Biologics Inc (Beijing, China).

Clean reads were aligned to the sweet potato reference genome using HISAT2 (Ipomoea_batatas. Pasi3_v2. genome. Fa), the genome can be obtained from https://sweetpotato.com/download_genome.html. Qualimap was used to assess mapping efficiency to evaluate alignment quality. Gene expression levels were quantified in FPKM (fragments per kilobase of transcript fragments per million mapped reads) based on unique mapping reads. Sample relationships were evaluated by calculating Pearson correlation coefficients (visualized via correlation heat maps) and principal component analysis (PCA) for FPKM-normalized expression data. Raw read counts were used as input for differential expression analysis using DESeq2. The resulting P-value was adjusted using the Benjamini-Hochberg approach for controlling the false discovery rate. Genes with an adjusted P-value < 0.01 and fold change ≥2 found by DESeq2 were assigned as differentially expressed. Functional annotation and enrichment analysis of DEGs were performed based on Gene Ontology (GO), KEGG, and g: Profiler.

Weighted correlation network analysis (WGCNA) was constructed to identify candidate genes highly correlated with insect resistance. To ensure the network satisfied the scale-free topology assumption, the soft-thresholding power (β) was selected based on the criterion that the corresponding scale-free topology fit index (R²) reached at least 0.8. The soft-thresholding power was set to β = 15 for leaf selection and β = 17 for storage root selection to ensure the network achieved a scale-free topology fit index of R2 > 0.8 (**Supplementary Figure 1**). Modules were detected using the dynamic tree-cut method (minModuleSize = 30) and merged with a dissimilarity threshold of 0.25. Pearson correlation matrices (R ≥ 0.75, p ≤ 0.05) were used to establish relationships between feature genes and core metabolites, with the parameters set to default values. nodes represent genes or metabolites, and edges denote significant correlations. The correlation network was visualized using Cytoscape v3.10.1.

### 2.6 Metabolome analysis

The treated and untreated samples were taken at 0 h and 4 h, respectively, with three biological replicates set for each group. After vacuum freeze-drying, 50 mg of each freeze-dried sample was accurately weighed, and 1.0 mL of a methanol-acetonitrile-water (1:2:1) mixture was added, followed by vortexing for 30 s. Low-temperature grinding was performed using a ball mill (45 Hz) for 10 min, followed by ultrasonic extraction in an ice-water bath for 10 min. The samples were kept at -20 °C for 1 h and then centrifuged at 12,000 rpm for 15 min at 4 °C. A 300 μL aliquot of the supernatant was filtered through a 0.22 μm microporous membrane into 2 mL injection vials. A 10 μL aliquot of each sample was pooled to prepare quality control (QC) samples before UPLC-MS/MS analysis. Metabolite identification was performed using the GB-plant database. Qualitative identification was based on compound-specific Q1, Q3, and retention time (RT) information, together with database matching. Quantitative analysis was conducted using a triple quadrupole mass spectrometer operated in multiple reaction monitoring (MRM) mode. Metabolite identification confidence was defined according to MSI guidelines (Sumner et al., 2007): Level 1 (confirmed, with authentic standards); Level 2 (putatively annotated, database MS/MS matching without standards); Level 3 (putatively characterized, class-level assignment based on spectral features); and Level 4 (unknown, not matched to any known compound).

UPLC-ESI-MS/MS analysis was performed by Biologics using an ultra-performance liquid chromatography-electrospray ionization-tandem mass spectrometry system (UPLC-ESI-MS/MS), with a Waters Acquity I-Class PLUS UPLC and an Applied Biosystems QTRAP 6500+ mass spectrometer. The chromatographic column was a Waters HSS-T3 column (1.8 μm, 2.1 mm × 100 mm). Mobile phase A was ultrapure water containing 0.1% formic acid and 5 mM ammonium acetate, and mobile phase B was acetonitrile containing 0.1% formic acid. The gradient elution program was as follows: 98% A and 2% B were held for 1.5 min, linearly changed to 50% A and 50% B within 5.0 min, linearly changed to 2% A and 98% B within 9.0 min, and held for 1 min. The gradient was then returned to 98% A and 2% B within 1 min and equilibrated for 3 min. The flow rate was set to 0.35 mL/min, the column oven temperature was set to 50 °C, and the injection volume was 4 µL. The chromatographic effluent was alternately introduced into the ESI-triple quadrupole-linear ion trap mass spectrometry system.

Ion source parameters were as follows: ion source temperature, 550 °C; ion spray voltage, 5500 V in positive ion mode and -4500 V in negative ion mode; and ion source gas I (GSI), gas II (GSII), and curtain gas (CUR) pressures of 50, 55, and 35 psi, respectively. Collision-activated dissociation (CAD) was set to moderate intensity. Instrument tuning and mass calibration were performed using 10 μmol/L and 100 μmol/L polypropylene glycol solutions in QQQ and LIT modes, respectively. QQQ scans were performed in multiple-reaction monitoring (MRM) mode, with nitrogen used as the collision gas at moderate intensity. The declustering potentials (DP) and collision energies (CE) of each MRM ion pair were further optimized. The corresponding MRM ion pairs were monitored separately according to the metabolites eluted in each time period.

Based on the detected metabolites, principal component analysis (PCA) and Spearman correlation analysis were performed using the R package factoextra to evaluate the repeatability of biological replicates and quality control samples. Unit variance (UV) scaling was performed on the data, and cluster heatmap analysis was performed for all samples. VIP values from the OPLS-DA model, combined with P-value/FC from univariate analysis, were used to screen for differential metabolites. The screening criteria for metabolomic differential metabolites (DAMs) were VIP > 1, log2FC ≥ 1, and FDR < 0.05, where FDR (false discovery rate) represents the P-value adjusted for multiple comparisons using the Benjamini-Hochberg method to reduce the risk of false positives due to multiple comparisons. Metabolites were annotated using the KEGG (http://www.kegg.jp/kegg/compound/), HMDB (https://hmdb.ca/metabolites/) and LipidMaps database (https://lipidmaps.org/).

To classify differentially accumulated metabolites (DAMs) based on their accumulation patterns before and after *C. formicarius* feeding in different sweet potato varieties, K-means clustering analysis was performed. Prior to clustering, the metabolite abundance matrix was log2-transformed and subjected to row-wise Z-score normalization to ensure comparability among features. The optimal number of clusters was determined using the silhouette coefficient, with independent evaluations conducted for the leaf and storage root datasets.

For pathway-level integration, pathway co-enrichment analysis was performed separately for DEGs and DAMs based on the KEGG pathway database using the hypergeometric test. Multiple testing correction was applied using the Benjamini-Hochberg method to control the false discovery rate (FDR), and pathways with an FDR ≤ 0.05 were considered significantly enriched.

### 2.7 Quantitative real-time PCR verification

To validate the transcriptome sequencing results, 14 candidate genes identified by multi-omics analysis were selected for qRT-PCR verification, including six genes related to phenylpropanoid biosynthesis, two genes related to flavonoid biosynthesis, two genes related to α-linolenic acid metabolism, two genes related to cutin, suberin, and wax biosynthesis, and two WRKY transcription factor genes. Among these, the phenylpropanoid biosynthesis related genes comprised four upregulated genes from leaves and two upregulated genes from storage roots. The flavonoid biosynthesis related genes and WRKY transcription factor genes were upregulated in both storage roots and leaves, whereas the α-linolenic acid metabolism related genes were derived from storage roots, and the cutin, suberin, and wax biosynthesis related genes were derived from leaves. Quantitative reverse transcription PCR (qRT-PCR) was performed using gene-specific primers designed based on the transcriptome data and ChamQ Universal SYBR qPCR Master Mix (Nanjing Vazyme Biotech Co., Ltd.). Three biological replicates from independent plants and three technical replicates were used for each sample. *IbTUB* (g9986) was used as the internal reference gene. Primer specificity was verified by melting curve analysis and agarose gel electrophoresis. Relative gene expression levels were calculated using the 2^−ΔΔCT^ method. The primer sequences of the internal reference genes (*IbTUB*) and all target genes are shown in **Supplementary Table 1**.

### 2.8 Statistical analysis

All experiments were performed with at least three biological replicates, and the results are presented as the mean ± standard error (SE). Statistical analyses were conducted using SPSS 26.0 (IBM, Armonk, NY, USA) and GraphPad Prism 9.0 (GraphPad Software, San Diego, CA, USA). Differences between two groups were analyzed using an independent Student’s t-test. For data involving cultivar and treatment effects at multiple time points, two-way analysis of variance (ANOVA) was employed, with cultivar and treatment (or time, as appropriate) as fixed factors and repeated measurements as the within-subjects factor. For other datasets, one-way or two-way ANOVA was performed as appropriate, followed by *post hoc* analysis using Tukey-Kramer multiple comparison tests. Differences were considered statistically significant at P < 0.05.

## 3 Results

### 3.1 Resistance of sweet potato cultivars to *C. formicarius*

After field investigation of 100 sweet potato cultivars, leaf damage index, stem tip damage index, and storage root damage index were evaluated (**Supplementary Figure 2**). Based on the combined evaluation results, the less affected cultivar J2 was selected as the insect-resistant cultivar, and the more severely affected cultivar G12 was selected as the insect-susceptible cultivar for subsequent laboratory evaluation and multi-omics analysis.

The laboratory resistance assessment further validated the field results. Leaf feeding area measurements and the consumption weight of storage roots indicated that the leaf feeding area and storage root consumption weight increased progressively over time at 2, 4, 6, and 8 h post-exposure to adult *C. formicarius*, but two parameters were significantly lower in the J2 than in the G12 at each time point (**Figures 1A-C**). Storage root resistance assessment revealed that, after exposure to 50 adults and a 30-day development period, the number of newly emerged *C. formicarius* adults from J2 storage roots was significantly lower than that from G12 storage roots (P < 0.001). (**Figures 1D, E**). Phenotypic observations demonstrated that the surfaces of G12 storage roots were covered with feeding holes and tunnels and developed rot and a foul odor; by contrast, damage to J2 storage roots was significantly less severe, with only a few signs of local feeding. These results indicated that both leaves and storage roots of J2 exhibited stable and significant pest resistance.

### 3.2 Transcriptome data quality assessment and quality control analysis

**Figure 2:**
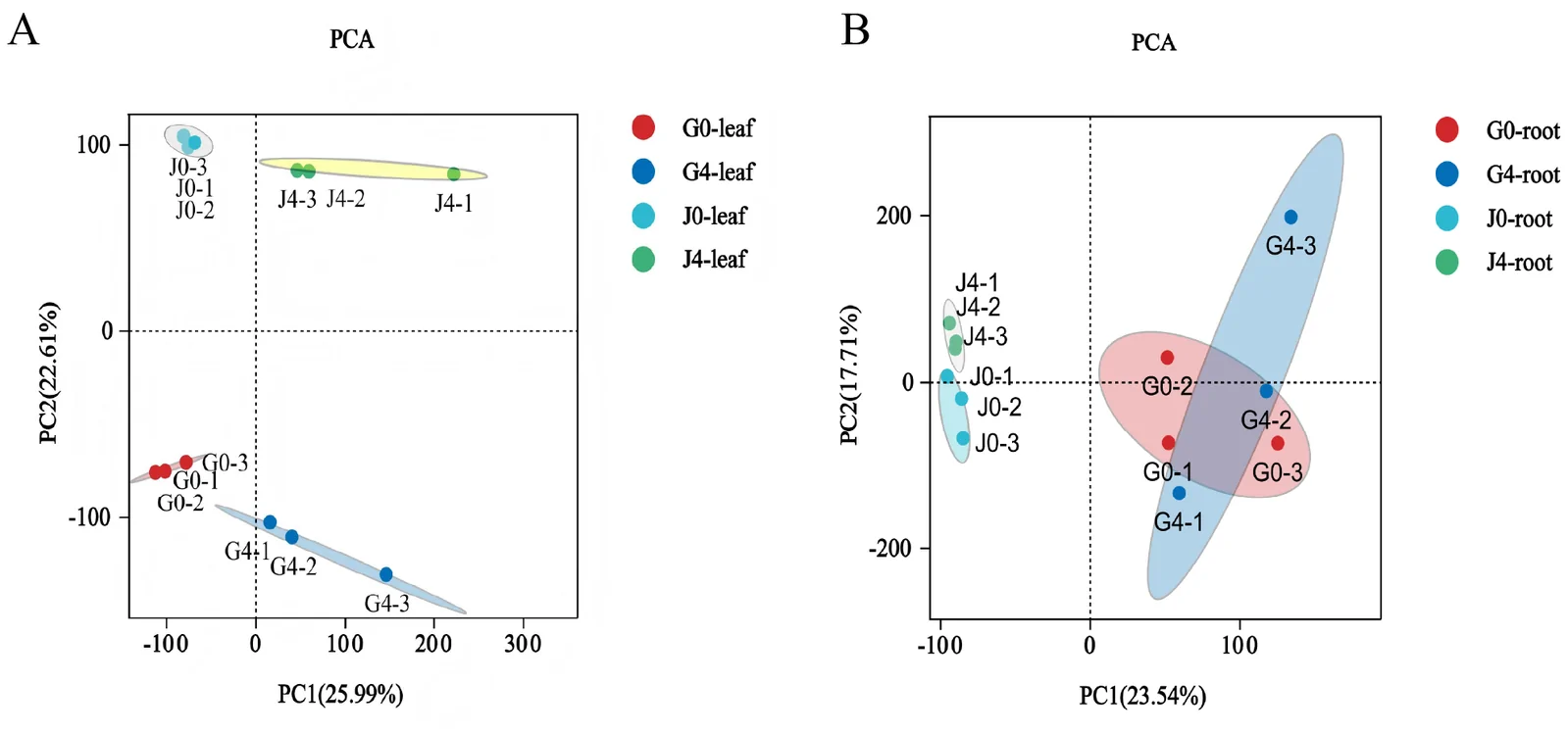
PCA of transcriptome samples. **(A)** Leaf transcriptome samples. **(B)** Storage root transcriptome samples.

Transcriptome sequencing was performed on sweet potato leaves and storage roots with and without *C. formicarius* feeding. After strict quality control, each sample produced 5.72-5.94 Gb of high-quality clean reads, with a total data volume of 153.61 Gb. The GC content of each sample was 45.84%-49.01%, and the percentage of Q30 bases was higher than 94.08% (**Supplementary Table 2**). The alignment rates of reads from each sample to the sweet potato reference genome (Ipomoea_batatas.pasi3_v2.genome.fa) ranged from 74.61% to 83.56% (**Supplementary Table 3**). Principal component analysis (PCA) results showed that the first principal component (PC1) and the second principal component (PC2) accounted for 25.99% and 22.61% of the overall variation, respectively, indicating a clear distinction between J2 and G12 (**Figures 2A, B**). These results indicated that the sequencing data from this study were of high quality and suitable for subsequent bioinformatics analysis.

### 3.3 Metabolomics analysis of sweet potato leaves in response to feeding stress by *C. formicarius*

**Figure 3:**
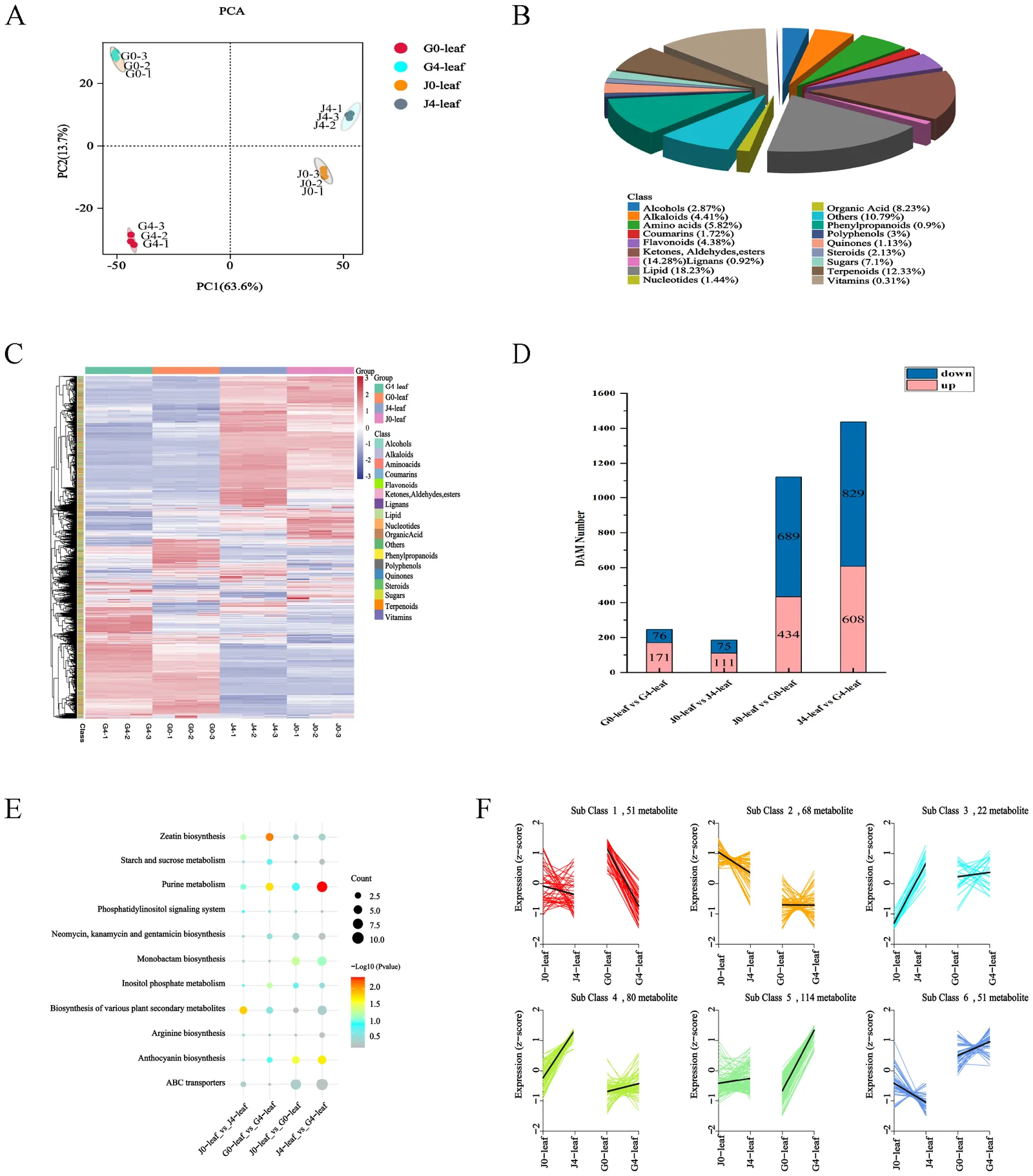
Metabolomic changes in sweet potato leaves in response to *C. formicarius* feeding stress. **(A)** PCA plot. **(B)** Metabolite classification statistics. **(C)** Dynamic changes in differential metabolites; heatmap shows relative metabolite abundance (red, upregulation; blue, downregulation). **(D)** Statistics of upregulated and downregulated DAMs of the two materials before and after feeding stress. The red column indicated the number of upregulated metabolites, and the blue column indicated the number of downregulated metabolites. **(E)** KEGG enrichment of leaf DAMs; dot size indicates number of metabolites, and color indicates enrichment significance. **(F)** K-means clustering of differential metabolites; X-axis represents feeding time points (0 h and 4 h); Y-axis standardized metabolite score; colored lines, expression dynamics of each metabolite; black lines, representative cluster expression. DAMs were identified using OPLS-DA models with criteria of VIP > 1, log2FC ≥ 1, and FDR < 0.05.

To identify metabolic changes in leaves following infestation, UPLC-MS/MS analysis was performed on J2 and G12 samples at 0 h and 4 h of feeding. Principal component analysis (PCA) showed that the quality control samples clustered closely, confirming the stability of the LC-MS system. Biological replicates within each group also clustered closely, indicating high repeatability, whereas different groups were clearly separated, suggesting significant differences in metabolite accumulation patterns between the two cultivars during herbivory (**Figure 3A**). A total of 3,900 metabolites were annotated and classified into 18 categories, including 711 lipids, 557 ketones, aldehydes, and esters, 481 terpenoids, 321 organic acids, 277 sugars, 227 amino acids, 172 alkaloids, 171 flavonoids, 117 polyphenols, 112 alcohols, 83 steroids, 36 lignans, 67 coumarins, 44 quinones, 35 phenylpropanoids, and 421 other metabolites (**Figure 3B**, **Supplementary Table 4**).

Comparative metabolomic analysis of J2 and G12 identified 386 differentially accumulated metabolites (DAMs) based on the screening criteria of VIP > 1, log2FC ≥ 1, and FDR < 0.05. Defense-related metabolites accumulated significantly in sweet potato leaves after 4 h of feeding stress, suggesting that sweet potato can rapidly initiate metabolic responses during the early stage of feeding (**Figure 3C**). In J2 leaves, 186 DAMs (111 upregulated and 75 downregulated) were identified at 4 h of feeding stress compared with the control. In G12 leaves, 247 DAMs (171 upregulated and 76 downregulated) were identified at 4 h compared with the control (**Figure 3D**). KEGG enrichment analysis indicated that, after feeding stress, DAMs in J2 leaves were mainly enriched in the “biosynthesis of plant secondary metabolites” pathway, whereas DAMs in G12 leaves were mainly enriched in pathways such as “zeatin biosynthesis” and “purine metabolism” (**Figure 3E**).

To further analyze the metabolic changes during the feeding period of *C. formicarius*, the K-means clustering algorithm was used to divide 386 differentially accumulated metabolites into six distinct clusters (**Supplementary Table 5**). Analysis of the six clusters revealed that two specific clusters (clusters 3 and 4) were observed to accumulate in large quantities in J2, while no significant changes were observed in G12. Among them, lipid, terpene, and organic acid metabolites demonstrated higher accumulation levels in J2 after 4-hour feeding treatment. This distinct accumulation pattern is associated with the stronger resistance phenotype of J2, suggesting that these specific metabolic clusters, particularly lipids, terpenoids, and organic acids, may contribute to defense against *C. formicarius* herbivory. In contrast, the absence of such accumulation in G12 may partly explain its susceptibility, consistent with a potential role for the ability to rapidly activate specific metabolic pathways in contributing to resistance in sweet potato. (**Figure 3F**, **Supplementary Table 6**).

### 3.4 Transcriptomic analysis of the response of sweet potato leaves to feeding stress by *C. formicarius*

**Figure 4:**
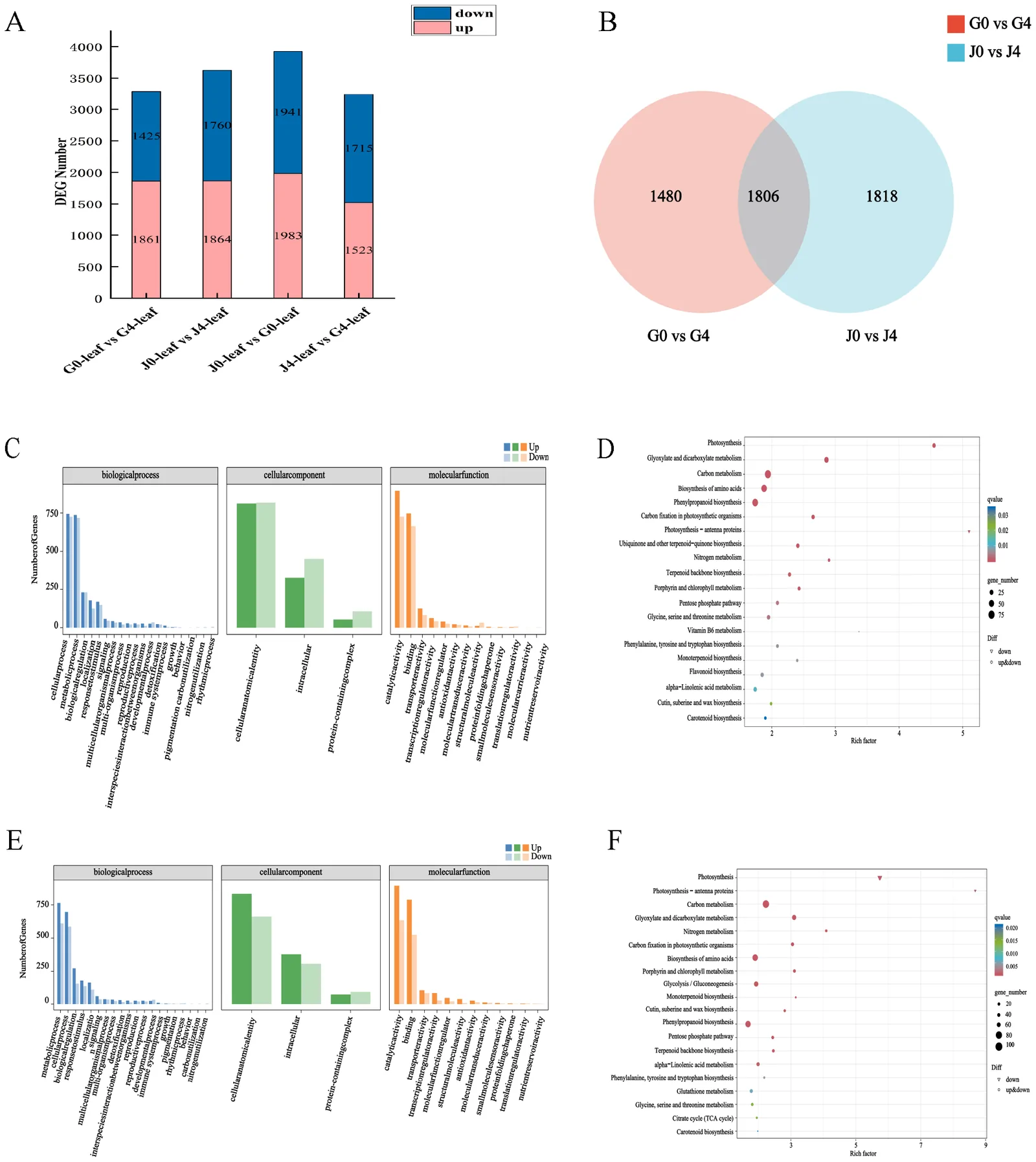
Transcriptome changes in sweet potato leaves in response to *C. formicarius* feeding stress and functional annotation of DEGs. **(A)** Number of DEGs identified in J2 and G12 leaves before (0 h) and after (4 h) feeding stress (fold change ≥2, adjusted P < 0.01). **(B)** Venn plot analysis of leaf DEGs in the two cultivars. **(C)** GO functional classification of DEGs in J2 leaves. **(D)** KEGG enrichment analysis of DEGs in J2 leaves. **(E)** GO functional classification of DEGs in G12 leaves. **(F)** KEGG enrichment analysis of DEGs in G12 leaves. The size of the dots in **(D, F)** indicates the number of genes, and color indicates enrichment significance. Statistical significance was determined based on an adjusted p-value (padj) threshold of < 0.05.

Leaf gene expression in J2 and G12 under no-feeding conditions (J0 and G0) and after 4 h of feeding (J4 and G4) was compared. In J2, a total of 3,624 DEGs were identified 4 h after feeding compared with the non-feeding control, including 1,864 upregulated and 1,760 downregulated genes. In G12, a total of 3,286 DEGs were identified 4 h after feeding, including 1,861 upregulated and 1,425 downregulated genes (**Figure 4A**). Further comparison of transcriptional differences in key metabolic pathways between the two cultivars revealed that, under herbivory, J2 had 1,818 unique DEGs, and G12 had 1,480 unique DEGs, with 1,806 DEGs shared by the two cultivars (**Figure 4B**). Overall, the number of DEGs identified in J2 leaves was slightly higher than that in G12, suggesting that J2 may exhibit a stronger transcriptional response during the early stage of feeding.

To further analyze the biological functions of DEGs, GO and KEGG enrichment analyses were performed. GO functional annotation showed that all DEGs could be classified into three major categories: biological process, cellular component, and molecular function. Among them, biological process terms were significantly enriched, with cellular process and metabolic process terms being dominant (**Figures 4C, E**). KEGG enrichment analysis indicated that DEGs in J2 leaves before and after feeding were significantly enriched in carbon metabolism, amino acid biosynthesis, and phenylpropanoid biosynthesis (**Figure 4D**). Genes differentially expressed in G12 leaves before and after feeding were significantly enriched in carbon metabolism, amino acid biosynthesis, and glycolysis/gluconeogenesis (**Figure 4F**). To verify the reliability of the leaf transcriptome data, four genes related to phenylpropanoid biosynthesis, two genes related to flavonoid biosynthesis, two genes related to cutin, suberin, and wax biosynthesis, and two WRKY transcription factor genes were selected for qRT-PCR validation. The qRT-PCR results were highly consistent with the RNA-seq data for the selected genes, confirming the reliability of the expression trends observed in our transcriptomic analysis (**Supplementary Figure 3**).

### 3.5 Combined analysis of leaf transcriptomics and metabolomics

**Figure 5:**
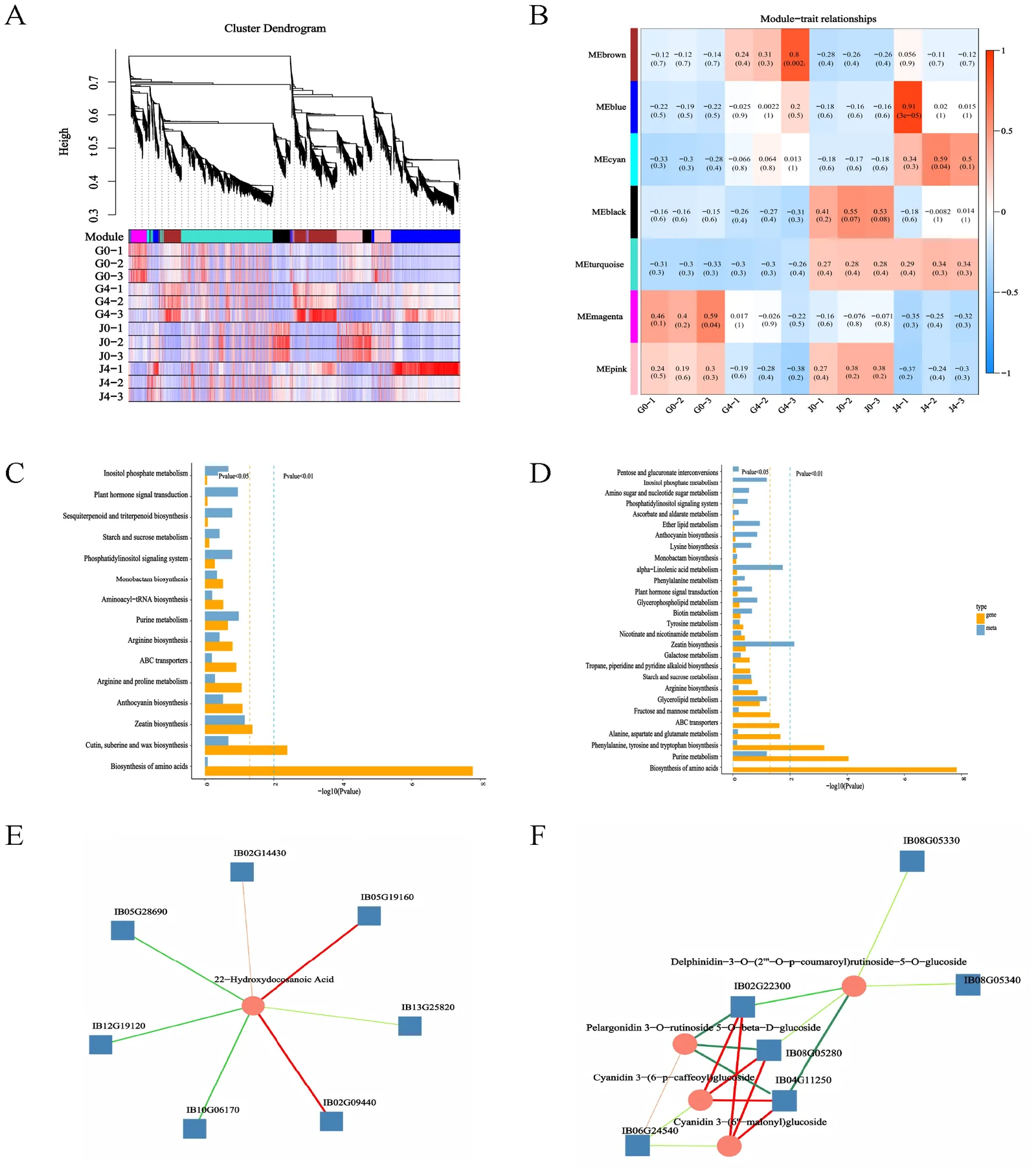
Combined transcriptome and metabolome analysis (leaves). **(A)** Co-expression module cluster tree. **(B)** Module-trait correlation heatmap. **(C)** Bar chart of common enrichment pathways in J2 leaves. **(D)** Bar chart of common enrichment pathways in G12 leaves. **(E)** Correlation network for cutin, suberin, and wax biosynthesis. **(F)** Correlation network for anthocyanin biosynthesis. Circles represent metabolites, boxes represent genes, and values on the lines represent correlation coefficients. Positive correlations are red, negative correlations are green, and greater correlation coefficients are indicated by wider and darker lines.

WGCNA was performed on all DEGs to identify key modules involved in the response to feeding stress and to gain a deeper understanding of the molecular mechanisms by which sweet potato responds to feeding. Based on similar expression patterns, a total of seven co-expression modules were identified in sweet potato leaves (**Figure 5A**). Module-trait association analysis showed that most DEGs in J2 leaves were concentrated in the MEcyan module, which was positively correlated with feeding treatment; in contrast, most DEGs in G12 leaves were mainly assigned to the MEbrown module, which was positively correlated with feeding treatment (**Figure 5B**). The co-enriched KEGG pathway bar graph of the two omics datasets showed significant enrichment in the “amino acid biosynthesis”, “cutin, suberin, and wax biosynthesis”, and “anthocyanin biosynthesis” pathways in J2 leaves after *C. formicarius* feeding stress (**Figure 5C**). G12 leaves showed significant enrichment in “purine metabolism”, “phenylalanine, tyrosine, and tryptophan biosynthesis” and “glycerolipid metabolism” pathways after *C. formicarius* feeding stress (**Figure 5D**). To further reveal the regulatory relationship between J2 leaf genes and metabolites, a correlation network for cutin, suberin, and wax biosynthesis and anthocyanin biosynthesis was constructed based on the expression data of DEGs and corresponding metabolites in the MEcyan module (**Figures 5E, F**).

### 3.6 Metabolomics analysis of the response of sweet potato storage roots to feeding stress by *C. formicarius*

**Figure 6:**
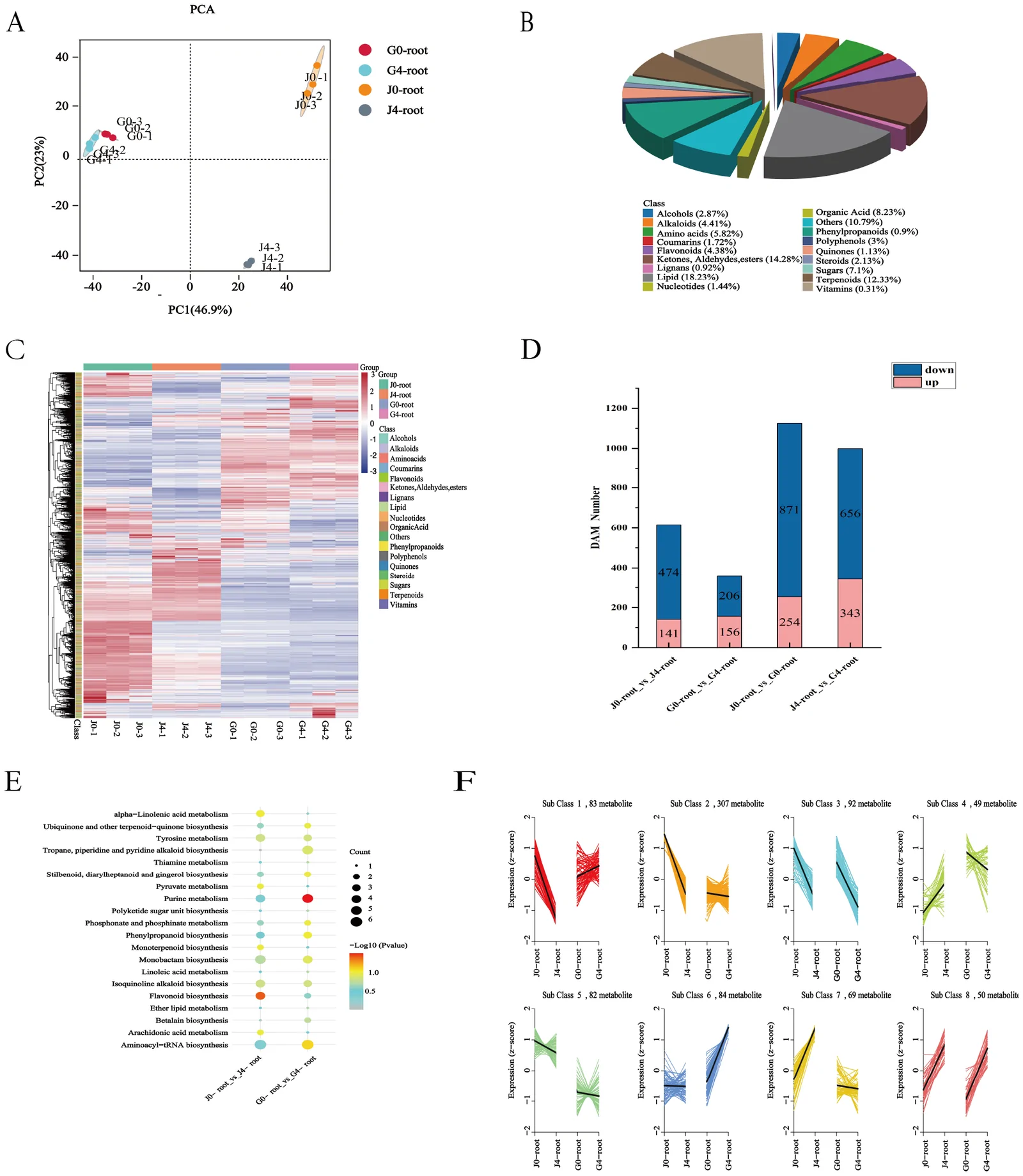
Metabolomic changes in sweet potato storage roots in response to *C. formicarius* feeding stress. **(A)** PCA plot. **(B)** Metabolite classification statistics. **(C)** Dynamic changes in differential metabolites; the heatmap shows relative metabolite abundance (red, upregulation; blue, downregulation). **(D)** Statistics of upregulated and downregulated DAMs of the two materials before and after feeding stress. The red column indicated the number of upregulated metabolites, and the blue column indicated the number of downregulated metabolites. **(E)** KEGG enrichment of storage roots DAMs; dot size indicates number of metabolites, and color indicates enrichment significance. **(F)** K-means clustering of differential metabolites; X-axis represents feeding time points (0 h and 4 h); Y-axis standardized metabolite score; colored lines, expression dynamics of each metabolite; black lines, representative cluster expression. DAMs were identified using OPLS-DA models with criteria of VIP > 1, log2FC ≥ 1, and FDR < 0.05.

To investigate metabolic changes in sweet potato storage roots under *C. formicarius* feeding stress, samples were collected at 0 h and 4 h after feeding treatment and analyzed using ultra-performance liquid chromatography-tandem mass spectrometry (UPLC-MS/MS). Principal component analysis (PCA) showed that the quality control samples clustered closely, indicating LC-MS system stability. Biological replicates within each group also clustered closely, indicating high repeatability, whereas the samples from different groups were significantly separated, indicating significant differences in metabolite accumulation patterns between J2 and G12 under attack (**Figure 6A**). A total of 3,900 metabolites were annotated and classified into 18 categories, including 711 lipids, 557 ketones, aldehydes, and esters, 481 terpenoids, 321 organic acids, 277 sugars, 227 amino acids, 172 alkaloids, 171 flavonoids, 117 polyphenols, 112 alcohols, 83 steroids, 36 lignans, 67 coumarins, 44 quinones, 35 phenylpropanoids, and 421 other metabolites (**Figure 6B**, **Supplementary Table 7**). Although a similar number of annotated metabolites were detected in both tissues, the major differences between leaf and storage root were reflected in their relative abundance profiles rather than in the total number of detected metabolites.

Comparative metabolomic analysis of J2 and G12 identified 816 differentially accumulated metabolites (DAMs) based on the screening criteria of VIP > 1, log2FC ≥ 1, and FDR < 0.05. Various defense-related metabolites in sweet potato storage roots changed significantly after 4 h of feeding stress, suggesting that sweet potato can rapidly initiate metabolic responses during the early stage of feeding (**Figure 6C**). In J2 storage roots, 615 DAMs (141 upregulated and 474 downregulated) were identified at 4 h of feeding stress compared with the control. In G12 storage roots, 362 DAMs (156 upregulated and 206 downregulated) were identified at 4 h compared with the control (**Figure 6D**). Statistical comparisons of differential metabolites showed that 161 DAMs changed significantly in both resistant and susceptible cultivars after *C. formicarius* feeding, indicating that these metabolites may play a fundamental role in the response of sweet potato storage roots to *C. formicarius* feeding stress (**Figure 6D**). KEGG enrichment analysis demonstrated that, after feeding stress, DAMs in J2 storage roots were mainly enriched in “flavonoid biosynthesis”, “α-linolenic acid metabolism,” and “monoterpene biosynthesis,” whereas DAMs in G12 storage roots were mainly enriched in “purine metabolism” and “aminoacyl-tRNA biosynthesis” (**Figure 6E**).

To further analyze metabolic changes during *C. formicarius* feeding, the K-means clustering algorithm was used to divide 816 differentially accumulated metabolites into 8 clusters (**Supplementary Table 8**). Analysis of the 8 clusters revealed that two specific clusters (clusters 4 and 7) accumulated at high levels in J2 and decreased in G12 under *C. formicarius* treatment. Lipid, terpene, flavonoid, and organic acid metabolites showed higher accumulation levels in J2 after 4 h of feeding treatment. This distinct accumulation pattern is associated with the stronger resistance phenotype of J2, suggesting that these specific metabolic clusters, particularly lipids, terpenoids, flavonoids, and organic acids may contribute to defense against *C. formicarius* herbivory in storage roots. In contrast, the absence of such accumulation or the observed decrease in G12 may partly explain its susceptibility; further supporting the hypothesis that the ability to rapidly activate and accumulate specific metabolite clusters under feeding stress is a key determinant of resistance in sweet potato storage roots (**Figure 6F**, **Supplementary Table 9**).

### 3.7 Transcriptomic analysis of the response of sweet potato storage roots to feeding stress by *C. formicarius*

**Figure 7:**
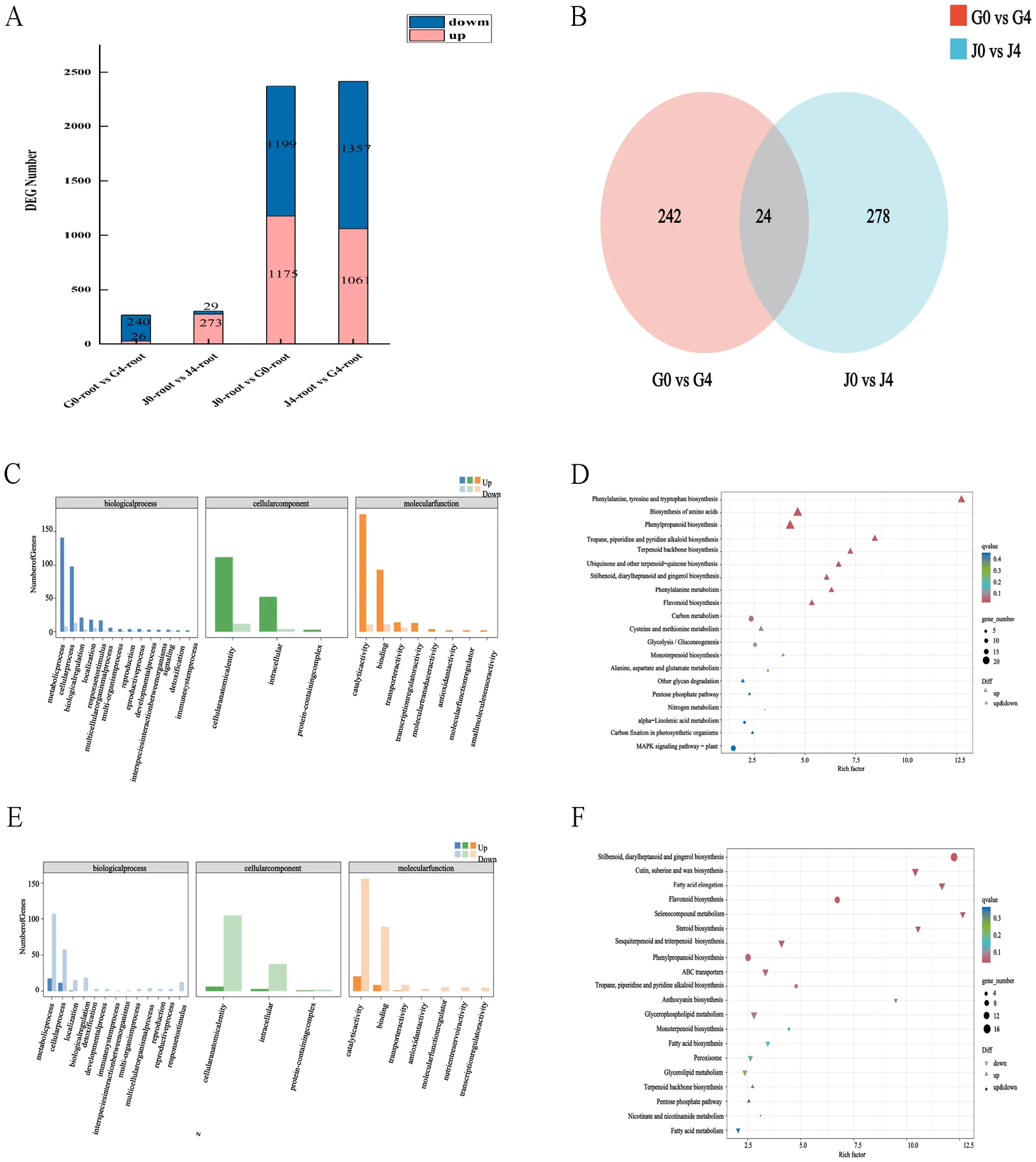
Transcriptome changes in sweet potato storage roots in response to *C. formicarius* feeding stress and functional annotation of DEGs. **(A)** Number of DEGs identified in J2 and G12 roots before (0 h) and after (4 h) feeding stress (fold change ≥2, adjusted P < 0.01). **(B)** Venn plots of J2 and G12 storage root DEGs. **(C)** GO functional classification of J2 storage root DEGs. **(D)** KEGG enrichment analysis of DEGs in J2 storage roots. **(E)** GO functional classification of DEGs in G12 storage roots. **(F)** KEGG enrichment analysis of G12 storage root DEGs. **(D, F)** The size of the dots indicates the number of genes, and color indicates enrichment significance. Statistical significance was determined based on an adjusted p-value (padj) threshold of < 0.05.

In J2 storage root sample, a total of 302 DEGs were identified after 4 h of feeding treatment compared with the non-feeding control group, including 273 upregulated genes and 29 downregulated genes. In G12 storage root sample, 266 DEGs were identified under the same treatment, including 26 upregulated and 240 downregulated genes (**Figure 7A**). Further comparisons of transcriptional differences in key metabolic pathways between the two cultivars in response to feeding stress showed that J2 had 278 specific DEGs, G12 had 242 specific DEGs, and 24 DEGs were shared by the two cultivars (**Figure 7B**). Under feeding stress, the number of DEGs identified in J2 was slightly higher than that in G12, and J2 was dominated by upregulated DEGs, whereas G12 was dominated by downregulated DEGs, suggesting that the two cultivars may adopt different transcriptional regulatory strategies under early feeding stress.

To analyze the biological functions of DEGs, GO and KEGG enrichment analyses were performed. GO classification showed that all DEGs were distributed among three major categories: biological process, cellular component, and molecular function. Biological process terms were significantly enriched, with metabolic process and cellular process terms being dominant (**Figures 7C, E**). DEGs in J2 storage roots before and after feeding were significantly enriched in “phenylpropanoid biosynthesis”, “phenylalanine, tyrosine, and tryptophan biosynthesis”, and “amino acid biosynthesis” (**Figure 7D**). DEGs in G12 storage roots before and after feeding were significantly enriched in “stilbenoid, diarylheptanoid, and gingerol biosynthesis”, “selenocompound metabolism”, and “steroid biosynthesis” (**Figure 7F**). To verify the reliability of the storage roots transcriptome data, two genes related to phenylpropanoid biosynthesis, two genes related to flavonoid biosynthesis, two genes related to α-linolenic acid metabolism, and two WRKY transcription factor genes were selected for qRT-PCR validation. The qRT-PCR results were highly consistent with the RNA-seq data for the selected genes, confirming the reliability of the expression trends observed in our transcriptomic analysis (**Supplementary Figure 4**).

### 3.8 Combined analysis of root transcriptomics and metabolomics

**Figure 8:**
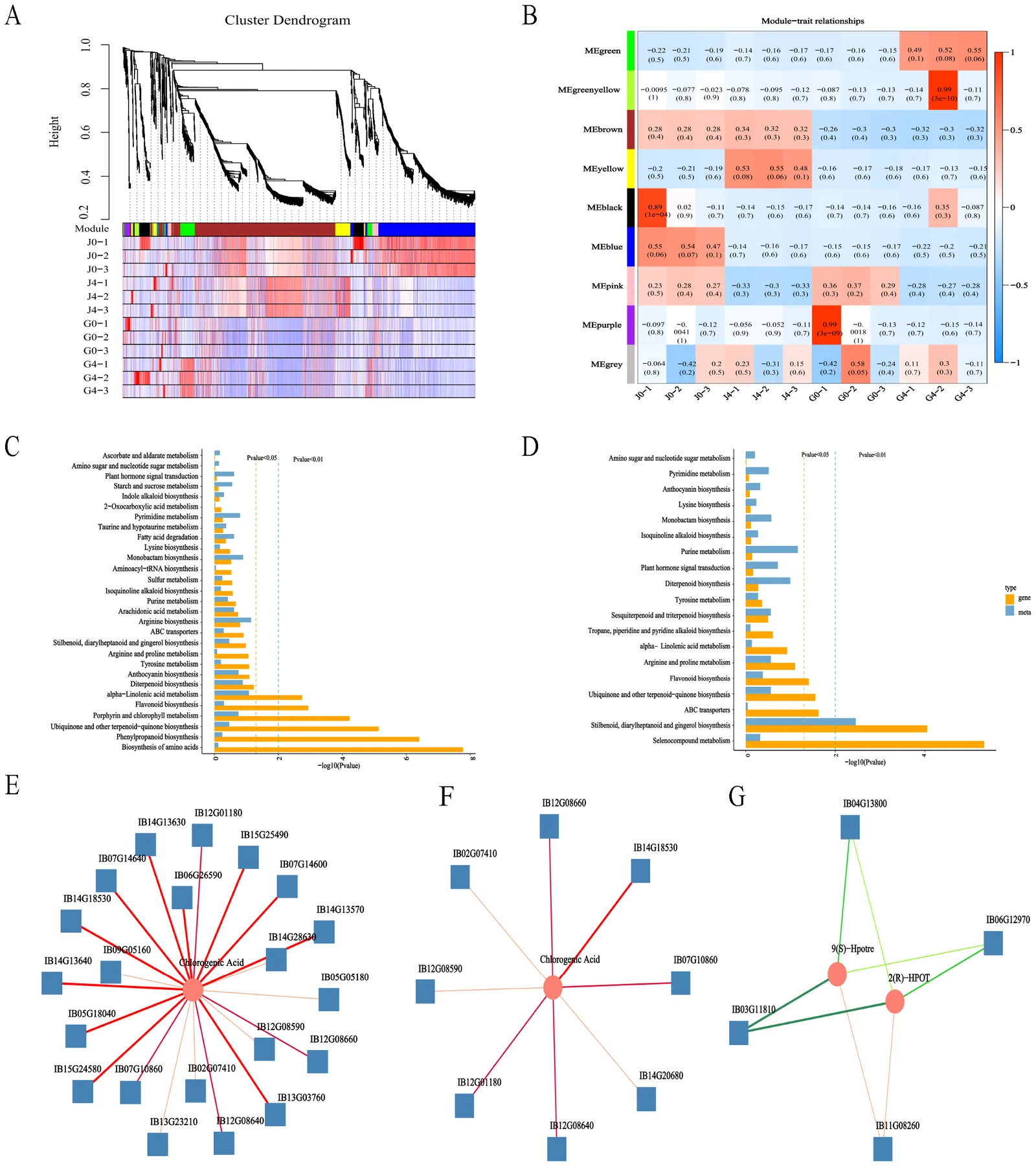
Combined transcriptome and metabolome analysis (storage roots). **(A)** Co-expression module cluster tree. **(B)** Module-trait correlation heatmap. **(C)** Bar chart of common enrichment pathways in J2 storage roots. **(D)** Bar chart of common enrichment pathways in G12 storage roots. **(E)** Correlation network for phenylpropanoid biosynthesis. **(F)** Correlation network for flavonoid biosynthesis. **(G)** Correlation network for α-linolenic acid metabolic pathway. Circles represent metabolites, boxes represent genes, and values on the lines represent correlation coefficients. Positive correlations are red, negative correlations are green, and greater correlation coefficients are indicated by wider and darker lines.

To further analyze the differential response mechanisms of sweet potato to *C. formicarius* feeding, WGCNA was conducted on all DEGs to identify potential key modules in response to feeding stress. Based on similar expression patterns, a total of 11 co-expression modules were identified in sweet potato storage roots (**Figure 8A**). Most DEGs in J2 storage roots were concentrated in the MEgreen module, whereas most DEGs in G12 storage roots were mainly assigned to the MEred module (**Figure 8B**). The co-enriched KEGG pathway bar graph of the two omics datasets showed significant enrichment in “phenylpropanoid biosynthesis”, “flavonoid biosynthesis”, and “α-linolenic acid metabolism” pathways in J2 storage roots after *C. formicarius* feeding stress (**Figure 8C**). G12 storage roots showed significant enrichment in “stilbenoid, diarylheptanoid, and gingerol biosynthesis” and “selenocompound metabolism” pathways after *C. formicarius* feeding stress (**Figure 8D**). To further reveal the regulatory relationship between J2 storage root genes and metabolites, a correlation network diagram of phenylpropanoid biosynthesis, flavonoid biosynthesis, and α-linolenic acid metabolism was constructed based on the expression data of DEGs and corresponding metabolites in the MEgreen module (**Figures 8E–G**).

## 4 Discussion

### 4.1 Differential responses of susceptible sweet potato cultivars to feeding stress by *C. formicarius*

Sweet potato weevils are devastating pest for global sweet potato production, their larvae and adults cause direct damage to storage roots and leaves, seriously restricting yield and quality (Xu et al., 2022). Therefore, analyzing sweet potato defense mechanisms against this pest is of great theoretical significance for breeding insect-resistant cultivars and developing green control strategies (Liu et al., 2022; Yada et al., 2023; Keyser et al., 2024). Through field resistance evaluation and indoor feeding experiments, this study confirmed that J2 demonstrated significantly lower damage and feeding preference in both leaves and storage roots than G12. Integrated transcriptomic and metabolomic analyses further revealed that the two cultivars exhibited organ- and developmental stage-specific transcriptional and metabolic responses to *C. formicarius* feeding. In storage roots, J2 showed more DEGs and DAMs than G12, indicating stronger induced defense activation; however, in leaves, J2 had more DEGs but fewer DAMs, suggesting a reliance on rapid transcriptional reprogramming rather than massive metabolite accumulation against adult feeding. These tissue-specific patterns demonstrate that J2 possesses enhanced transcriptional and metabolic remodeling capacities overall, with their expression being differentially regulated across organs. This phenomenon is consistent with the “induced defense advantage” commonly observed in studies of plant pest resistance: pest-resistant genotypes can rapidly activate defense signaling networks during the early stage of pest invasion, thereby effectively limiting pest feeding and tissue damage (Erb and Reymond, 2019; Lee et al., 2025; Xu et al., 2026).

A significant difference in the number of DEGs was observed between leaves and storage roots in J2 (3,624 in leaves vs. 302 in storage roots), suggesting organ-specific defense strategies. Leaves, which are directly fed on by adults, must quickly sense mechanical damage and elicitors in oral secretions and therefore exhibit broader and stronger transcriptional responses. Storage roots, as underground storage organs, face continuous larval feeding and must also balance energy storage and developmental regulation. Their defense responses therefore tend toward focused activation, with efficient coordination among a limited number of core pathways (Vasantha-Srinivasan et al., 2025).

Although G12 also produced more DEGs in leaves, its enrichment pathways were more concentrated in processes related to growth regulation or basal metabolism, such as zeatin biosynthesis and purine metabolism, and the accumulation of defensive metabolites was less extensive and persistent than that of J2. This pattern suggests that G12 may show a typical “growth-defense trade-off” bias: it fails to shift sufficient resources toward efficient defense when stress occurs and instead maintains strong growth- or repair-related metabolism (Li et al., 2024; Zaret et al., 2024).

### 4.2 Phenylpropanoid and flavonoid metabolic pathways form the core metabolic hubs of pest resistance in sweet potato

The phenylpropanoid metabolic pathway is a core network of plant secondary metabolism and provides synthetic precursors for a variety of defensive compounds, such as lignin, phenolic acids, flavonoids, and anthocyanins (Jiang et al., 2024). This study found that J2 was significantly enriched in the “phenylpropanoid biosynthesis” and “flavonoid biosynthesis” pathways in both leaves and storage roots under feeding stress, and the expression levels of related genes and metabolites were significantly higher in J2 than in G12. These findings are consistent with reports from multiple crops in recent years. For example, phenylpropanoid and flavonoid metabolic remodeling in tea plants during aphid feeding is considered an important component of the defense response (Jiang et al., 2024). In wild relatives of tomato, combined transcriptomic and metabolomic analyses showed that enhanced phenylpropanoid biosynthesis could increase aphid resistance, and silencing *Sl4CLL6* reduced downstream *HCT*, *CAD*, and *CHI* expression and weakened resistance (Wang et al., 2024). In maize, salt-tolerant cultivars significantly accumulate flavonoids such as dihydroquercetin and apigenin by upregulating key flavonoid synthesis genes such as *ZmCHI6*, thereby enhancing reactive oxygen species scavenging capacity and membrane stability (Wang et al., 2025). In rice, *OsCHI3* enhances drought tolerance by synergistically regulating flavonoid and abscisic acid metabolic pathways (Liu, 2025). In addition, flavonoid metabolites accumulated significantly in J2 storage roots in this study. Flavonoids have been shown to deter feeding, inhibit digestive enzyme activity, and interfere with insect growth and development.

Notably, the “cutin, suberin, and wax biosynthesis” pathway was simultaneously enriched in J2 leaves, suggesting that J2 employs a dual strategy of “physical barrier + chemical defense” to effectively prevent *C. formicarius* from feeding. In contrast, this pathway was not significantly enriched in G12, further supporting the importance of physical epidermal barrier reinforcement in the resistant phenotype. Future studies using tissue sectioning, lignin/xylem staining, wax content determination, and functional verification of key structural genes are needed to confirm the specific roles of cutin, suberin, and wax metabolism in the resistance response (Zhu et al., 2023; Jin et al., 2025).

### 4.3 Amino acid metabolism and the α-linolenic acid/JA signaling pathway work in tandem to drive the defense response

Amino acid metabolism not only provides raw materials for protein synthesis but also serves as a precursor source for a variety of secondary metabolites, such as phenols, alkaloids, and nitrogen-containing defense compounds (Rahim et al., 2023). The “amino acid biosynthesis” pathway was significantly enriched in both J2 leaves and storage roots, suggesting that J2 provides sufficient substrate supply for downstream defense pathways by enhancing amino acid metabolism. In particular, phenylalanine is a direct substrate of the phenylpropanoid pathway, and increased metabolic flux through phenylalanine may be a key limiting step in secondary metabolic activation in J2.

Meanwhile, the α-linolenic acid metabolic pathway was significantly enriched in the J2 storage roots, but not in the leaves. α-linolenic acid is a direct precursor of JA biosynthesis, and its significant enrichment suggests that the JA-associated signaling pathway may be involved in the response of sweet potato to the feeding stress of *C. formicarius*. JA is a core signaling molecule for plants in response to chewing insect pests and mechanical damage, inducing the expression of various defense proteins (such as protease inhibitors, polyphenol oxidase) and secondary metabolites (Wasternack and Song, 2017; Xing et al., 2024; Marques-Galvez et al., 2025). Similarly, in potatoes, Sweet11-mediated glucose signaling enhanced the JA-mediated resistance response by degrading two JA-induced susceptibility factors, MCPI1a and MCPI1b (Mao et al., 2025). In quinoa, two MYB transcription factors, *CqMYB44L1* and *CqMYB44L2*, were rapidly induced by *Spodoptera exigua* feeding, and their overexpression could increase JA content and grant JA-mediated resistance. The *CqMYB44* protein was shown to bind to the promoter of JA synthesis-related genes and activate their expression (Liu et al., 2025). In tomatoes, *SlWRKY81* synergistically regulates spermidine synthesis and Na^+^/K^+^ homeostasis by interacting with the key factor of the JA pathway, *SlJAZ1*, enhancing tolerance to saline-alkali stress (Shang et al., 2024). In maize, *ZmWRKY82* enhances saline-alkali tolerance by binding to the W-box element of the *ZmCHI6* promoter to activate flavonoid synthesis and eliminate reactive oxygen species (Wang et al., 2025). Based on these results, we hypothesize that in sweet potato storage roots, enhanced α-linolenic acid metabolism induced by *C. formicarius* feeding might activate JA signaling, which then regulates the accumulation of flavonoid and phenylpropanoid defense products through transcription factors such as WRKY, MYB, or bHLH, suggesting that sweet potato may have a defense response pattern involving both signal regulation and metabolic reprogramming (Ho et al., 2020; Pastierovic et al., 2024b). Further research is necessary to verify through experiments such as exogenous JA treatment, JA synthesis inhibitors, and gene silencing or overexpression genetic transformation of key transcription factors.

### 4.4 Organ and developmental stage-specific defense responses of leaves and storage roots

This study revealed pronounced differences between leaves and storage roots in response to *C. formicarius* feeding. However, these differences may be influenced by both organ and developmental stage-specific. Leaves exhibited substantially more differentially expressed genes (DEGs) and a broader range of responsive pathways than storage roots, whereas storage roots showed fewer DEGs but more concentrated defense-related responses, particularly those associated with jasmonic acid (JA) signaling. These contrasting patterns may be explained by the distinct biological roles of the two organs, as well as by their developmental context. As aboveground organs, leaves are directly exposed to herbivory and environmental fluctuations and therefore may require rapid, broad-spectrum, and reversible defense responses. In contrast, storage roots, as underground reserve organs, must balance defense with storage function. Because excessive defense activation may impose a substantial growth–defense trade-off, storage roots may adopt a more economical yet efficient strategy by allocating resources to a limited number of key metabolic pathways (Monson et al., 2022).

K-means clustering analysis further supported these distinct response patterns. In the leaves, 386 DAMs were divided into six clusters, among which clusters 3 and 4 (containing lipids, terpenoids, and organic acids) accumulated significantly over feeding time in J2 but showed no significant change in G12. In storage roots, 816 DAMs were divided into 8 clusters, among which clusters 4 and 7 (containing lipids, terpenoids, flavonoids, and organic acids) also accumulated strongly in J2 but decreased in G12. These results suggest that J2 can specifically activate the synthesis and accumulation of specific metabolite clusters during herbivory, whereas these metabolite clusters showed a weak response in G12. Similar organ-dependent defense differentiation has been reported in other plant systems. For example, under saline–alkali stress in maize, roots primarily regulate ion transport and cell wall remodeling, whereas leaves preferentially enhance antioxidant capacity and osmotic adjustment (Cao et al., 2020; Wang et al., 2025).

Importantly, in the present study, leaf samples were collected from 30-day-old seedlings, whereas storage roots were sampled from 120-day-old plants. The developmental difference between sampled organs may also influence the magnitude and nature of defense responses. Mature storage roots contain abundant stored carbohydrates and pre-existing secondary metabolites, which may allow them to rely more on constitutive or metabolite-based defense mechanisms rather than extensive transcriptional activation. Conversely, young leaves are actively growing tissues with highly dynamic transcriptional activity and may exhibit stronger inducible responses following insect attack. Therefore, the observed differences between leaves and storage roots likely reflect the combined effects of organ specialization, developmental status, and ecological function.

Our findings suggest that the defense patterns of leaves and storage roots are shaped by both organ identity and developmental stage. Future studies using developmentally matched sampling or stage-controlled comparisons will be necessary to disentangle the respective contributions of organ type and developmental status, thereby enabling a more precise understanding of defense regulation in sweet potato under *C. formicarius* attack.

### 4.5 WGCNA identifies hub modules and candidate genes of the insect-resistant regulatory network

Weighted gene co-expression network analysis (WGCNA) is a powerful tool for mining gene modules and core regulators associated with complex traits (Peng et al., 2021). WGCNA identified the MEcyan module in leaves and the MEgreen module in storage roots; both modules were significantly positively correlated with insect resistance. These modules were significantly enriched in pathways such as phenylpropanoid metabolism and amino acid metabolism and contained multiple key genes for flavonoid synthesis (such as *CHS*, *CHI*, and *F3H*) and transcription factors (such as WRKY, MYB, and bHLH). Genes in these modules may be involved in the coordinated regulation of sweet potato responses to *C. formicarius* feeding, providing a reference for subsequent screening of candidate genes related to insect resistance.

Compared with traditional single-gene differential expression analysis, WGCNA can better reveal groups of co-expressed genes and potential hub regulators under complex stress conditions. For example, in salt-tolerant maize studies, WGCNA closely associated the MEgreen module with flavonoid metabolites and *ZmWRKY82*, which was subsequently confirmed to directly activate *ZmCHI6* expression (Wang et al., 2025). Similarly, the MEcyan and MEgreen modules identified in this study provide a high-confidence candidate gene pool for subsequent functional validation. Future work should prioritize the most highly connected transcription factors and core genes from these modules and validate their functions using qRT-PCR, virus-induced gene silencing (VIGS), overexpression, genetic transformation, and metabolite remodeling.

**Figure 9:**
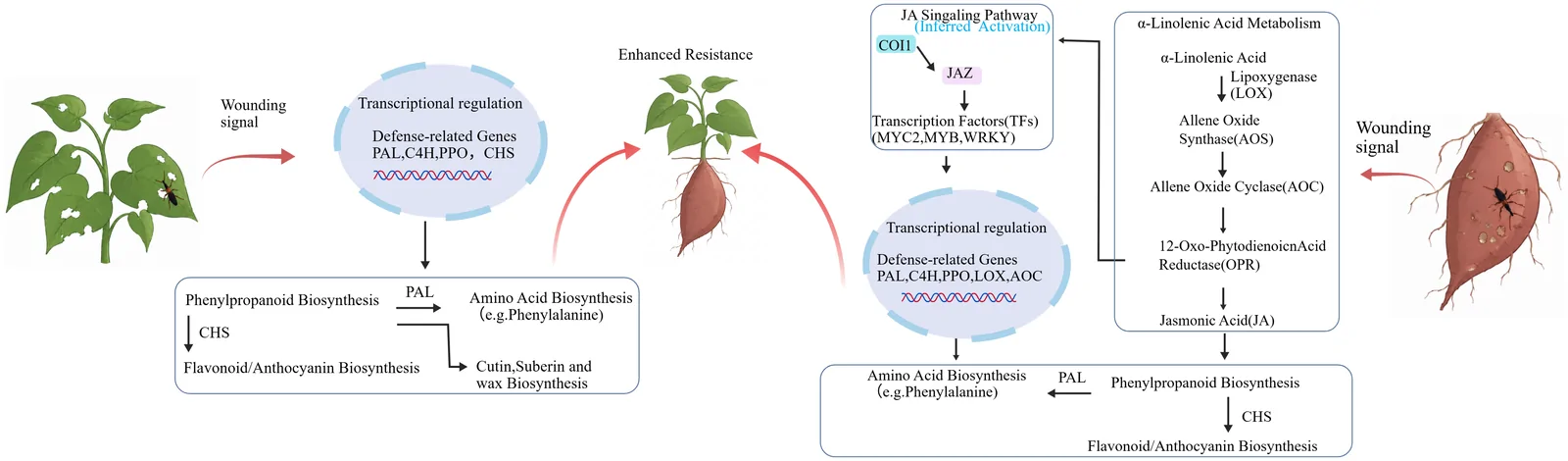
Schematic model of sweet potato defense responses to *C. formicarius* herbivory. In leaves, wounding signals induced by *C. formicarius* feeding activate the transcription of defense-related genes, including *PAL*, *C4H*, *PPO*, and *CHS*, thereby promoting phenylpropanoid biosynthesis, flavonoid accumulation, amino acid biosynthesis, and the formation of cutin, suberin, and wax. In storage roots, herbivory-triggered wounding signals stimulate α-linolenic acid metabolism and jasmonic acid (JA) biosynthesis through the LOX–AOS–AOC–OPR pathway. JA signaling is subsequently transduced through the COI1–JAZ module and downstream transcription factors, such as MYC2, MYB, and WRKY, leading to the activation of defense-related genes, including *PAL*, *C4H*, *PPO*, *LOX*, and *AOC*. These coordinated responses contribute to enhanced resistance against *C. formicarius*.

Based on the integrated transcriptomic and metabolomic results, we propose a working model for sweet potato defense against *C. formicarius* herbivory (**Figure 9**). In this model, herbivory-induced wounding activates α-linolenic acid metabolism and JA signaling, which subsequently regulate defense-related transcription factors and metabolic pathways, ultimately reinforcing both chemical and physical defense barriers.

Despite these insights, this study focuses on early defense responses (0–4 h). Dynamic changes over longer time courses and the functional validation of candidate hub genes (e.g., via VIGS or overexpression) remain to be investigated in future work.

## 5 Conclusions

This study integrated transcriptomic and metabolomic analyses to compare the early-stage responses of the resistant cultivar J2 and the susceptible cultivar G12 to *C. formicarius* herbivory. The results revealed that J2 exhibited strong early transcriptional and metabolic responses in both leaves and storage roots, and the identified DEGs and DAMs were primarily enriched in pathways such as phenylpropanoid biosynthesis, flavonoid biosynthesis, amino acid metabolism, cutin, suberin, and wax biosynthesis, and α-linolenic acid metabolism. Combined multi-omics analysis and WGCNA results suggest that phenylpropanoid metabolism, amino acid metabolism, and several secondary-metabolism-related modules may be involved in the defense response of sweet potato to *C. formicarius* feeding stress. Overall, this study provides a reference for understanding the molecular and metabolic basis of insect resistance in sweet potato and a basis for subsequent functional validation of candidate genes and metabolites related to insect resistance.

## Data Availability

The datasets presented in this study can be found in online repositories. The names of the repository/repositories and accession number(s) can be found in the article/**Supplementary Material**.
