# Supplementary material for: Integrated transcriptomic and metabolomic analyses of early defense responses in resistant and susceptible sweet potato cultivars under Cylas formicarius herbivory
Source: Front Plant Sci. 2026 Aug 18;17:1901251. doi: 10.3389/fpls.2026.1901251 (PMC13531670; doi:10.3389/fpls.2026.1901251)
Supplement: Supplementary file 1 [file DataSheet1.docx]

Supplementary Material

# 1 Surveys

## 1.1 Uganda Survey

For this study, a detailed survey was conducted during the dry season months of early 2023 (with initial pilot data collection in December 2022, and main national data collection from late Jan to late April, 2023). The survey covered rural areas with cropland, but excluded rural areas without cropland and urban areas. The total area for the sampled areas visited by Columbia World Projects (CWP) enumerators is equivalent to 83,431 sq km, or ~75% of the national area with farmland, or ~42% of the total national land area. The CWP survey interviewed 13,840 farmers, between 0.2 and 0.3% of estimated rural households.

**Supplementary Figure 1:** *Sample Areas in Uganda that were surveyed. Green dots indicate SAs where at least 1 irrigating farmer was found and red indicate SAs where no irrigation was found.*

From the survey work and interviews with farmers in Uganda certain limitations of utilizing remote sensing methods for irrigation detection emerged. A lot of these farmlands are smaller than the pixel resolution of Sentinel 2 which is 10 m thus making vegetation patterns harder to detect due to the mixed signal. Limited knowledge and access to resources also contributes to adding to the challenge of detecting irrigation. Several farmers tend to grow multiple crops within their agriculture plot which is aimed at serving subsistence farming needs. Proper irrigation knowledge and resources are either limited or absent (*Participatory research and development with smallholder farmers in Uganda to improve small-scale irrigation*, no date). The result of which is not knowing when to irrigate and the water requirements for crop growth. This is evident from surveys that showed that a lot of these farmers tend to use manual methods such as carrying water using handheld containers to their farms to irrigate. Some key observations were:

- 88% manual irrigation, 9% fuel pumps, 1% solar pumps
- 85% handheld containers, 11% pipes/hoses, 3% trench canals for water transport
- 73% surface water, 9% boreholes, 11% shallow wells
- 90% Irrigating farmers grew their crops to be sold

 **Supplementary Figure 2:** *Images from surveys show how people carry water to fields manually or use surface water paired with diesel pumps to irrigate their fields.*

## 1.2 Zambia Survey

The Quadracci Sustainable Engineering Lab team conducted a survey in 2024 from May to August in Zambia to understand where farmers are irrigating, resulting in irrigation data collected for 95 mini-grid sites. The sites are distributed in the following provinces:

- Eastern (49)
- North-Western (13)
- Southern (17)
- Muchinga (8)
- Central (5)
- Northern (5)
- Western (1)

This resulted in a sparse dataset of farm plot coordinates that utilize irrigation. Farmers employed low cost methods of enhancing groundwater recharge by digging trenches next to the fields without added cost of drilling boreholes.

**Supplementary Figure 3:** *Distribution of mini-grid sites across Zambia. Most of these site visits are in the Eastern province with some across the North0Western, Southern and Muchinga provinces,*

# 2 Input Features for the model

A list of input features used in the feature study is provided in Supplementary Table 1. Supplementary Figure 4 provides a correlation matrix among these features. This allows us to eliminate features that are highly correlated with each other. For example BIO1, LSTN and LSTD are highly correlated and only one of these features can be used without loss of much information contained within them. Similarly BIO15, DIPA and DNLT are correlated with each other suggesting the distance to protected areas and night lights are related to the precipitation seasonality. PARA EVI SOC, SOC and elevation are correlated showing the soil organic content is related to the annual EVI and elevation. Net productivity annual (NPPA) and seasonal (NPPS) indicators are highly correlated with each other for the irrigated and non-irrigated locations

**Supplementary Table 1:** List of Input Features for irrigation detection

| Feature Code | Description | Category | Data Source |
| --- | --- | --- | --- |
| EVI | Enhanced Vegetation Index | Vegetation | Sentinel-2 / MODIS |
| HAND | Height Above Nearest Drainage | Topographic | HydroSHEDS / SRTM |
| SRTMmtpi | Multi-Scale Topographic Position Index |  | SRTM 30m DEM |
| elevation | Raw elevation above sea level. |  | SRTM DEM |
| slope | Terrain slope calculated from DEM |  | SRTM 30m DEM |
| TIM | Topographic Index Map |  | DEM Derived |
| CTI | Compound Topographic Index |  | DEM Derived |
| LSTD / LSTN | Average Land Surface Temperature (Daytime / Nighttime) (degC) | Climatic | MODIS |
| BIO1 / BIO7 | Mean Annual Temperature / Temperature Range (degC * 10) |  | WorldClim |
| BIO12 / BIO15 | Mean Annual Precipitation / Precipitation Seasonality (mm/yr, CV) |  | WorldClim |
| PARA/PARV | fAPAR (Average/Variance) |  | MODIS |
| NPPA / NPPS | Net Primary Productivity (Annual / Seasonal) |  | MODIS |
| DOWS | Distance to Surface Water | Anthropic | Global Surface Water (GSW) |
| DHRSL | Distance to High-Resolution Settlements |  | Meta/CIESIN HRSL |
| DNLT | Distance to Night Lights (km) |  | VIIRS |
| DOR1 / DOR2 | Distance to Roads (Major/ all) (km) |  | GRIP, OpenStreetMap |
| DIPA / DNPA | Distance to Internationally/ Nationally Protected Areas (km) |  | WDPA |
| SOC | Predicted top Soil Organic Carbon content (g/kg) | Soil | SoilGrids / AfSIS |
| PH | Predicted top soil pH in water (0-20 cm) |  | SoilGrids / AfSIS |
| CEC | Predicted top soil cation exchange capacity (cmol/kg) |  | SoilGrids / AfSIS |
| LS | Calculated slope length factor in the Revised Universal Soil Loss Equation |  | DEM Derived |
| SND | Sand Content: Describes soil drainage and texture. |  | SoilGrids / AfSIS |
| lat / lon | Latitude and Longitude of the farm plot. | Spatial | GPS Coordinates |
| LCCS | Land Cover Classification System |  | FAO |

**Supplementary Figure 4:** *Correlation Matrix of features. High values indicate represent high collinearity within the features. Such features are excluded from the classification models.*

As described in the methodology section, we capture the effect of the longitude feature which relates to the distinct phenological cycles in each region by ranking the important features by region. Northern Uganda (West Nile, Acholi and Karamoja subregions) is dominated by a single cropping cycle, Southern Uganda (Western, South Western, Central 1and 2 East Central ) by dual cropping cycle and Central Uganda (Lango, Tesso and Elgon subregions) consists of a mixture of the two. A beeswarm plot broken down by each region is shown in Figure 5.

**Supplementary Figure 5:** *SHAP beeswarm plot showing distributions of feature values for top 20 features ranked for a catboost model broken down by each region in Uganda.*

**Supplementary Figure 6:** *Scatter Plot comparing ERA5-Land 2023 climatic variables to Worldclim V1 BIO variables for Uganda. A 1:1 line is shown to facilitate comparison.*

**Supplementary Figure 7:** *Violin plots of feature values for irrigated and non-irrigated croplands shown. Distributions in light pink correspond to non-irrigated croplands and in light blue are irrigated croplands. The plot at the bottom lists some of the features that were analyzed while the bottom plot corresponds to the comparison of the Sentinel 2 EVI values across the year from January 0_EVI to December 11_EVI and also the annual mean EVI.*

# 3 Spatial blocks for cross-validation

**Supplementary Figure 8:** *Spatial blocks/Grids for cross-validation shown on the left and mask created to generate pseudo-absence points shown on the right*

Supplementary Figure 8 shows the 50km x 50km grids on the left that were used for spatial block cross validation approach. Using a 70-30 split, training data is randomly sampled from 70% of the grids and the performance is assessed for testing data sampled from the other 30% of the grids. This is repeated 5 times for the methodology. On the right of Figure 8 is the mask created to sample points for non-irrigated. The mask is generated by using a 5km threshold on the existing points. Apart from augmenting the existing non-irrigated dataset, this ensures that other non-cultivated land cover such as water bodies, forests etc. is included in the non-irrigated dataset.

# 4 Phenology Maps

**Supplementary Figure 9:** *Scatter plot for red, green and blue band values (from left to right) from the phenology maps of Uganda corresponding to single cropping cycles, evergreen vegetation and dual cropping cycles respectively.*

# 5 Extended Results

**Supplementary Table 2:** Performance metrics for different pseudo absence sampling strategies for Uganda

| Pseudo Absence Sampling Method | F1 score | Precision | Recall | Accuracy |
| --- | --- | --- | --- | --- |
| Cropland mask | 0.792 | 0.851 | 0.741 | 0.812 |
| 5km Buffer | 0.808 | 0.873 | 0.753 | 0.827 |

**Supplementary Table 3:** Performance metrics for different model runs for Zambia

| Method | Sampling | Run | F1 score | Precision | Recall | Accuracy |
| --- | --- | --- | --- | --- | --- | --- |
| AEF Embeddings | 10m | 1 | 0.923 | 0.989 | 0.865 | 0.896 |
|  |  | 2 | 0.958 | 0.944 | 0.972 | 0.957 |
|  |  | 3 | 0.925 | 0.968 | 0.886 | 0.902 |
|  |  | Average | 0.935 | 0.967 | 0.908 | 0.918 |
|  | 20m | 1 | 0.913 | 0.981 | 0.855 | 0.884 |
|  |  | 2 | 0.954 | 0.952 | 0.956 | 0.954 |
|  |  | 3 | 0.942 | 0.962 | 0.924 | 0.923 |
|  |  | Average | 0.937 | 0.965 | 0.911 | 0.920 |
|  | 30m | 1 | 0.937 | 0.964 | 0.911 | 0.913 |
|  |  | 2 | 0.953 | 0.927 | 0.981 | 0.952 |
|  |  | 3 | 0.930 | 0.935 | 0.925 | 0.906 |
|  |  | Average | 0.940 | 0.942 | 0.939 | 0.923 |
| Feature Engineering | 10m | 1 | 0.905 | 0.957 | 0.858 | 0.870 |
|  |  | 2 | 0.924 | 0.986 | 0.869 | 0.927 |
|  |  | 3 | 0.939 | 0.957 | 0.922 | 0.918 |
|  |  | Average | 0.922 | 0.967 | 0.883 | 0.905 |
|  | 20m | 1 | 0.937 | 0.959 | 0.917 | 0.912 |
|  |  | 2 | 0.944 | 0.979 | 0.912 | 0.946 |
|  |  | 3 | 0.927 | 0.933 | 0.922 | 0.901 |
|  |  | Average | 0.936 | 0.957 | 0.917 | 0.920 |
|  | 30m | 1 | 0.923 | 0.940 | 0.908 | 0.893 |
|  |  | 3 | 0.967 | 0.962 | 0.972 | 0.967 |
|  |  | 3 | 0.936 | 0.905 | 0.970 | 0.911 |
|  |  | Average | 0.942 | 0.935 | 0.950 | 0.924 |

# References

Participatory research and development with smallholder farmers in Uganda to improve small-scale irrigation (no date) Innovations in Dry Season Horticulture. Available at: https://www.hortirrigation.org/.
